# Association between Prenatal Lead Exposure and Blood Pressure in Children

**DOI:** 10.1289/ehp.1103736

**Published:** 2011-09-27

**Authors:** Aimin Zhang, Howard Hu, Brisa N. Sánchez, Adrienne S. Ettinger, Sung Kyun Park, David Cantonwine, Lourdes Schnaas, Robert O. Wright, Hector Lamadrid-Figueroa, Martha Maria Tellez-Rojo

**Affiliations:** 1 Department of Environmental Health Sciences, University of Michigan School of Public Health, Ann Arbor, Michigan, USA; 2 Department of Environmental Health, Harvard School of Public Health, Boston, Massachusetts, USA; 3 Channing Laboratory, Department of Medicine, Brigham and Women’s Hospital, Harvard Medical School, Boston, Massachusetts, USA; 4 Department of Biostatistics, University of Michigan School of Public Health, Ann Arbor, Michigan, USA; 5 Division of Chronic Disease Epidemiology, Yale Schools of Public Health and Medicine, New Haven, CT, USA; 6 Department of Epidemiology, University of Michigan School of Public Health, Ann Arbor, Michigan, USA; 7 Division of Public Health, National Institute of Perinatology, Mexico City, Distrito Federal, Mexico, USA; 8 Department of Emergency Medicine, Children’s Hospital, Harvard Medical School, Boston, Massachusetts, USA; 9 Division of Statistics, Center for Evaluation Research and Surveys, National Institute of Public Health, Cuernavaca, Morelos, Mexico

**Keywords:** blood pressure, children, lead, prenatal exposure, sex

## Abstract

Background: Lead exposure in adults is associated with hypertension. Altered prenatal nutrition is associated with subsequent risks of adult hypertension, but little is known about whether prenatal exposure to toxicants, such as lead, may also confer such risks.

Objectives: We investigated the relationship of prenatal lead exposure and blood pressure (BP) in 7- to 15-year-old boys and girls.

Methods: We evaluated 457 mother–child pairs, originally recruited for an environmental birth cohort study between 1994 and 2003 in Mexico City, at a follow-up visit in 2008–2010. Prenatal lead exposure was assessed by measurement of maternal tibia and patella lead using *in vivo* K-shell X-ray fluorescence and cord blood lead using atomic absorption spectrometry. BP was measured by mercury sphygmomanometer with appropriate-size cuffs.

Results: Adjusting for relevant covariates, maternal tibia lead was significantly associated with increases in systolic BP (SBP) and diastolic BP (DBP) in girls but not in boys (*p*-interaction with sex = 0.025 and 0.007 for SBP and DBP, respectively). Among girls, an interquartile range increase in tibia lead (13 μg/g) was associated with 2.11-mmHg [95% confidence interval (CI): 0.69, 3.52] and 1.60-mmHg (95% CI: 0.28, 2.91) increases in SBP and DBP, respectively. Neither patella nor cord lead was associated with child BP.

Conclusions: Maternal tibia lead, which reflects cumulative environmental lead exposure and a source of exposure to the fetus, is a predisposing factor to higher BP in girls but not boys. Sex-specific adaptive responses to lead toxicity during early-life development may explain these differences.

Events occurring in early life may affect cardiovascular health across the life course (Barker and Bagby 2005). For example, suboptimal growth *in utero* is associated with accelerated weight gain in children during childhood and greater risk of later hypertension, cardiovascular disease (CVD), and diabetes (Bagby 2007; Huxley et al. 2000). Little is known about how other prenatal insults, such as exposure to environmental toxicants, may also affect subsequent children’s risks of such conditions.

Lead is a ubiquitous environmental pollutant that accumulates in the human body, notably in bone, after exposure. Developmental toxicity of lead has recently emerged as a potentially large public health problem because substantial mobilization of maternal skeletal lead stores occurs during pregnancy (Hu 1998; Tellez-Rojo et al. 2004) and because the fetus is particularly vulnerable to environmental toxicants (Grandjean et al. 2008). Prenatal lead exposure has been linked, for example, to intrauterine growth restriction and neurodevelopmental toxicity (Cory-Slechta et al. 2008; Gomaa et al. 2002; Gonzalez-Cossio et al. 1997; Hernandez-Avila et al. 2002).

Although adult lead exposure is an established risk factor for hypertension and CVD in adults (Korrick et al. 1999; Nash et al. 2003; Navas-Acien et al. 2007; Weisskopf et al. 2009), less is known about the lead–blood pressure (BP) association in children (Chen et al. 2006; Factor-Litvak et al. 1996; Gump et al. 2005). Because childhood BP is an established precursor of hypertension and CVD in adults (Lauer and Clarke 1989), studying the relationship of early-life environmental exposures to childhood BP may shed light on the development of adult hypertension.

A previous longitudinal study reported a positive association between umbilical cord blood lead, a commonly used surrogate for prenatal exposure, and BP during childhood (Gump et al. 2005). However, the timing of fetal dose remains unclear, because cord lead mostly represents fetal exposure just before delivery and not throughout the entire pregnancy. Furthermore, although numerous studies have found sex differences in the association between markers of prenatal insults and BP (Gilbert and Nijland 2008; Jones et al. 2008; Loos et al. 2001; Taylor et al. 1997; te Velde et al. 2004), sex-specific susceptibility to lead in the developmental programming of cardiovascular regulation has not yet been examined. Indeed, gonadal hormones and sex-linked genes could affect any adaptive responses to lead through development and maturation and in later life (Jedrychowski et al. 2009).

In the present study, we capitalized on a long-running environmental birth cohort study to examine the relationship of prenatal lead exposure, assessed by both maternal bone and umbilical cord lead, with BP in 7- to 15-year-old children. We hypothesized that children of mothers with higher levels of lead accumulated in bone would have higher BP than would children of mothers with lower bone lead, and that this impact of lead on BP might differ according to child sex.

## Materials and Methods

*Study population.* Mother–child pairs in this study were drawn from three of the four longitudinal birth cohort studies in Mexico City that comprise the Early Life Exposures in Mexico to Environmental Toxicants (ELEMENT) project. Subjects were originally recruited between 1994 and 2003 to investigate the long-term consequences of prenatal exposure on child development (Gonzalez-Cossio et al. 1997; Tellez-Rojo et al. 2004). Detailed information on the study design and data collection procedures has been published previously (Ettinger et al. 2009; Gonzalez-Cossio et al. 1997; Hernandez-Avila et al. 2002). Briefly, baseline information on health status and on social and demographic characteristics was collected from all eligible participants at delivery and 1 month postpartum. Anthropometric data from the mother and newborn, and umbilical cord and maternal venous blood samples were gathered within 12 hr of delivery. Information on estimated gestational age, based on the date of last menstrual period, and characteristics of the birth and newborn period were extracted from the medical records. Maternal dietary energy, calcium, and iron intakes were calculated on the basis of a semiquantitative food-frequency questionnaire designed to estimate usual dietary intake over the prior month (Ettinger et al. 2009; Romieu et al. 1997). Interviewers explained the study to and obtained written consent from eligible women who were willing to participate and provided information on ways to minimize lead exposure. Exclusion criteria included factors that could interfere with maternal calcium metabolism; medical conditions that could cause low birth weight; prematurity (< 37 weeks) or an infant with Apgar score at 5 min of ≤ 6, a condition requiring treatment in neonatal intensive care unit; birth weight < 2,000 g, or serious birth defects; psychiatric illness, seizures, or kidney or cardiac disease; preeclampsia, systolic BP (SBP) > 140 mmHg or diastolic BP (DBP) > 90 mmHg; gestational diabetes; consumption of alcoholic beverages; addiction to illegal drugs; and continuous use of corticosteroids.

The mother–child pairs were contacted and recalled for the follow-up assessment between 2008 and 2010 when the children were 7–15 years of age. Only one child for each mother was included in this study, regardless of birth order. Medical history, physical examination, BP measurements, and venous blood samples were collected from or performed on mothers and children. Of 1,272 mother–child pairs who were eligible for this study, 631 (49.6%) attended the follow-up study evaluation. The most common reason for nonparticipation was the inconvenience of making the follow-up visit to the clinic. Of those participating in the follow-up visit, 457 (72.4%) completed the study with data on all variables of interest and were included in the analysis. At time of this visit, mothers and children old enough to provide assent to participate were given detailed information about present study procedures and signed a written letter of informed consent. The human subjects committees of all participating institutions approved this research.

*Lead measurements.* Maternal tibia (cortical) and patella (trabecular) bone lead levels were measured within 1 month of delivery using a spot-source ^109^Cd K-shell X-ray fluorescence (K-XRF) instrument (ABIOMED, Danvers, MA, USA) (Hu et al. 1998). Umbilical cord blood lead was analyzed using an atomic absorption spectrometry instrument (model 3000; PerkinElmer, Chelmsford, MA, USA), and child concurrent blood lead was analyzed using inductively coupled plasma mass spectrometry (Elan 6100; PerkinElmer, Norwalk, CT, USA). Because of the logistical constraints posed by the collection of samples during birth from multiple hospitals and at unpredictable hours, as well as subject’s concerns about venipuncture, we obtained data on lead levels in cord blood and current venous blood, respectively, from 323 (70.7%) and 367 (80.3%) of children participating in this study.

*Blood pressure end points.* At the follow-up assessment, BP was measured in both mothers and children. The mothers were instructed to abstain from smoking and from drinking alcohol and caffeine-containing beverages and children from drinking caffeine-containing beverages for at least 12 hr before coming to the study center. Immediately after the medical history review, while the subjects remained seated for an additional 5 min, the resting SBP and DBP of the mother and the child were measured by trained clinical personnel on the participant’s left arm using a standard mercury column sphygmomanometer and a cuff of appropriate size.

*Statistical analyses.* Statistical analysis was carried out using SAS (version 9.2; SAS Institute Inc., Cary, NC, USA). We examined univariate distributions of all variables and descriptive statistics and identified outliers before bivariate and multivariate analyses. Umbilical cord blood lead concentrations were transformed to their natural logarithmic values to normalize the right-skewed distribution. Differences between groups defined by child sex or missing cord lead were evaluated using *t*-tests for continuous variables and Pearson’s chi-square tests for categorical variables. Simple linear regression was used to quantify unadjusted associations between BP and cord or bone lead, as well as other covariates.

The adjusted associations between BP and cord or bone lead, as well as other covariates, were then assessed using multiple linear regression. Models for SBP and DBP were simultaneously fitted using generalized estimating equations to account for the correlation in these outcomes. Covariates were chosen based on biological plausibility or significant bivariate associations with BP. A base model was fitted that included maternal education (years), smoking (yes/no), dietary intakes of calories (kilocalories), calcium (milligrams), and iron (milligrams) during pregnancy; and infant gestational age (weeks) and weight (kilograms) at birth, birth order, sex, and child’s concurrent age (years), height (centimeters), and body mass index (BMI; kilograms per square meter). Each exposure variable was added to the base model one at a time, and models were run in the combined sample (boys and girls) as well as stratified by sex. The final models were fitted in the combined sample, and a cross-product term was introduced to evaluate the interaction of each lead exposure variable with sex. We also examined the potential confounding effect of current lead exposure by further adjusting for child’s blood lead level at the follow-up visit. Additive models with smoothed terms for exposure were also estimated to assess the nature of the association between lead exposure and BP. Partial residuals and estimated smoothed terms were plotted. Because bone lead is also known to be associated with higher BP in women (Korrick et al. 1999; Rothenberg et al. 2002), and maternal BP is a risk factor for child’s BP and therefore may be in the causal pathway, we did not include maternal BP as a standard covariate in the analyses. Instead, we conducted a sensitivity analysis by adjusting for maternal DBP, which is substantially heritable (Perusse et al. 1989; van Rijn et al. 2007) but relatively less affected by bone lead than is SBP (Navas-Acien et al. 2008).

## Results

Prenatal and later follow-up characteristics for mothers and their children are presented in Table 1. All of the 457 participant mothers were Mexican, with a mean (± SD) age of 25.6 ± 5.4 years (range, 19–31 years) at the time of the index child’s birth. The 1-month postpartum maternal tibia and patella bone lead had median [interquartile range (IQR)] values of 9.3 (3.3–16.1) and 11.6 (4.5–19.9) μg/g, respectively. Umbilical cord blood lead had a mean of 5.51 ± 3.45 μg/dL. The mean for available concurrent blood lead levels was 2.96 ± 1.72 μg/dL. Spearman correlations between any two exposure biomarkers ranged from 0.10 to 0.36 (*p* < 0.05). The mean age of children at follow-up was 10.7 ± 2.4 years (range, 7–15 years). The mean SBP and DBP were 94.9 ± 10.5 and 61.4 ± 8.2 mmHg, respectively. Of the 457 child participants, 46% were girls and 54% were boys. There were no significant differences between girls and boys for maternal bone or umbilical cord lead levels. Boys had slightly higher blood lead levels at the time of BP measurement than did girls (*p* = 0.10) (Table 1). Significant differences by sex, with higher mean values for boys, were observed for birth weight (*p* < 0.01) and height of children at follow-up (*p* < 0.01). Girls were, on average, shorter by 3 cm (Table 1). No other differences by sex were observed.

**Table 1:** Characteristics of the study participants, overall and by child sex.

Variable*a*	Overall (*n* = 457)	Girls (*n* = 211)	Boys (*n* = 246)	*p*-Value*b*
Maternal characteristics during index pregnancy								
Maternal age (years)		25.6 ± 5.4		25.7 ± 5.5		25.6 ± 5.3		0.56
Maternal education (years)		10.5 ± 3.0		10.3 ± 2.8		10.7 ± 3.2		0.39
Smoking during pregnancy (% yes)		3.5		4.3		2.8		0.16
Calories (kcal)		1.44 ± 0.58		1.45 ± 0.61		1.44 ± 0.56		0.75
Calcium (mg)		2,303 ± 708		2,313 ± 766		2,296 ± 657		0.80
Iron (mg)		20.7 ± 15.4		21.2 ± 15.2		20.2 ± 15.7		0.47
Tibia lead (μg/g)		9.3 (3.3–16.1)		9.99 (3.5–16.3)		9.1 (3.1–16)		0.33
Patella lead (μg/g)		11.6 (4.5–19.9)		10.8 (4.6–20.9)		12.2 (4.5–18.5)		0.92
Maternal characteristics during follow-up								
SBP (mmHg)		109.9 ± 11.3		109.2 ± 11		109.3 ± 11		0.32
DBP (mmHg)		72 ± 8.4		71.5 ± 8.3		72.3 ± 8.3		0.74
Child characteristics at birth								
Gestational age (weeks)		39 ± 1.3		38.9 ± 1.3		39.1 ± 1.3		0.69
Birth weight (g)		3,130 ± 432		3,066 ± 440		3,190 ± 418		< 0.01
Cord blood lead (μg/dL)*c*		5.51 ± 3.45		5.67 ± 3.67		5.48 ± 3.27		0.63
Child characteristics at follow-up								
Age (years)		10.7 ± 2.4		10.6 ± 2.4		10.8 ± 2.4		0.79
Birth order		2.04 ± 1.13		2.03 ± 113		2.05 ± 113		0.82
Height (cm)		143 ± 14		142 ± 12		145 ± 15.4		< 0.01
BMI (kg/m^2^)		20 ± 3.9		19.7 ± 3.7		20.2 ± 4.1		0.15
Blood lead (μg/dL)*d*		2.96 ± 1.72		2.75 ± 1.66		3.55 ± 1.76		0.10
SBP (mmHg)		94.9 ± 10.5		94.6 ± 10.5		95 ± 10.5		0.66
DBP (mmHg)		61.4 ± 8.2		60.7 ± 8.4		61.5 ± 8.2		0.52
**a**Values are median (IQR) for bone lead, percentage for smoking during pregnancy, and mean ± SD for all other variables. **b**Based on *t*-tests or Pearson’s chi-square tests. **c**Children with umbilical cord blood lead level, *n* = 323: 143 girls and 180 boys. **d**Children with concurrent blood lead level, *n* = 367: 176 girls and 191 boys.								

In the simple linear regression analyses of nonlead covariates, children’s age, height, and BMI were significantly associated with SBP or DBP in both girls and boys (Table 2). Tibia lead was significantly associated with SBP in girls, whereas cord blood lead was significantly associated with DBP in boys. Maternal age, dietary intake of calories, calcium, and iron during pregnancy, weight at birth, birth order, and child blood lead were not significantly associated with BP.

**Table 2:** Parameter estimate (mean ± SD) from simple linear regression of child BP in relation to maternal lead biomarkers and other factors.

SBP					DBP				
Variable	Overall (*n* = 457)	Boys (*n* = 246)	Girls (*n* = 211)	Overall (*n* = 457)	Boys (*n* = 246)	Girls (*n* = 211)
Maternal characteristics during index pregnancy												
Maternal age (years)		0.03 ± 0.09		0.03 ± 0.09		0.04 ± 0.09		–0.10 ± 0.1		–0.09 ± 0.1		–0.11 ± 0.1
Maternal education (years)		–0.12 ± 0.2		–0.10 ± 0.2		–0.16 ± 0.2		–0.17 ± 0.1		–0.14 ± 0.1		–0.24 ± 0.1
Smoking during pregnancy (% yes)		–0.91 ± 3.0		–0.86 ± 3.0		–1.01 ± 3.0		–2.0 ± 2.17		–1.88 ± 2.2		–2.27 ± 2.2
Calories (kcal)		0.58 ± 0.72		0.57 ± 0.75		0.59 ± 0.75		0.55 ± 0.50		0.70 ± 0.53		0.43 ± 0.52
Calcium (mg)		–0.67 ± 0.9		–0.62 ± 0.9		–0.75 ± 1.0		–0.39 ± 0.7		–0.17 ± 0.7		–0.71 ± 0.73
Iron (mg)		–0.16 ± 0.3		–0.05 ± 0.4		–0.28 ± 0.4		–0.09 ± 0.2		0.04 ± 0.27		–0.24 ± 0.25
Tibia lead (μg/g)		0.10 ± 0.0**		0.07 ± 0.06		0.13 ± 0.05**		0.05 ± 0.04		0.03 ± 0.05		0.07 ± 0.05
Patella lead (μg/g)		0.08 ± 0.04		0.08 ± 0.05		0.07 ± 0.05		0.04 ± 0.04		0.04 ± 0.04		0.026 ± 0.04
Maternal characteristics during follow-up												
SBP (mmHg)		0.32 ± 0.04		0.32 ± 0.04		0.32 ± 0.04		0.20 ± 0.03		0.20 ± 0.03		0.19 ± 0.03
DBP (mmHg)		0.38 ± 0.06		0.38 ± 0.06		0.38 ± 0.06		0.29 ± 0.05		0.29 ± 0.05		0.29 ± 0.05
Child characteristics at birth												
Gestational age (weeks)		0.30 ± 0.38		0.30 ± 0.38		0.30 ± 0.38		0.41 ± 0.31		0.41 ± 0.31		0.39 ± 0.31
Birth weight (g)		1.53 ± 1.28		1.53 ± 1.28		1.52 ± 1.35		1.3 ± 0.95		1.28 ± 0.95		1.09 ± 1.02
Birth order		0.05 ± 0.45		–0.13 ± 0.5		0.27 ± 0.50		–0.31 ± 0.3		–0.37 ± 0.4		–0.25 ± 0.38
Cord blood lead (μg/dL)		0.32 ± 0.18		033 ± 0.19		0.31 ± 0.22		0.26 ± 0.12**		0.28 ± 0.14**		0.24 ± 0.14
Child characteristics during follow-up												
Child age (years)		1.49 ± 0.18*		1.48 ± 0.19*		1.52 ± 0.19*		1.19 ± 0.15*		1.2 ± 0.15*		1.18 ± 0.16*
Child height (cm)		0.25 ± 0.03*		0.25 ± 0.03*		0.25 ± 0.03*		0.19 ± 0.02*		0.19 ± 0.03*		0.19 ± 0.03*
Child BMI (kg/m^2^)		1.01 ± 0.13*		1.0 ± 0.13*		1.02 ± 0.12*		0.61 ± 0.10*		0.62 ± 0.10*		0.60 ± 0.10*
Child blood lead (μg/dL)		0.20 ± 0.13		0.22 ± 0.14		0.15 ± 0.20		–0.01 ± 0.2		0.03 ± 0.14		–0.10 ± 0.22
*p < 0.001, **p < 0.05, derived from testing linear regression coefficients.												

In the base multivariate regression model (data not shown), with the combined sample, DBP was significantly associated with child age [0.75 mmHg; 95% confidence interval (CI): 0.14, 1.37 mmHg], BMI (0.37 mmHg; 95% CI: 0.16, 0.59 mmHg), and birth order (–0.66 mmHg; 95% CI: –1.26, –0.06 mmHg) and maternal education (–0.23 mmHg; 95% CI: –0.49, 0.02 mmHg). Although not statistically significant, the associations between SBP and age, birth order, and maternal education were in the same direction as those for DBP; SBP was significantly associated with child BMI (0.76 mmHg; 95% CI: 0.47, 1.05 mmHg).

Adjusted tibia, patella, and cord blood lead were not significantly associated with SBP (Table 3). However, in sex-stratified analyses, significant associations between tibia lead and SBP or DBP were observed only among girls (data not shown). In final models that formally tested sex differences using interaction terms, we found significant interactions between sex and tibia lead for child SBP and DBP (*p*-interaction = 0.025 and 0.006, respectively), indicating a stronger association of tibia lead with BP among females. Among girls, an IQR increase in maternal tibia lead (13 μg/g) was associated with 2.11-mmHg (95% CI: 0.69, 3.52 mmHg) and 1.60-mmHg (95% CI: 0.28, 2.91 mmHg) increases in SBP and DBP, respectively. In contrast, tibia lead tended to be nonsignificantly negatively associated with SBP (*p* = 0.68) and DBP (*p* = 0.18) among boys. The sex difference of the adjusted association between patella lead and BP was similar to that for tibia lead, but the relationship was not as strong or statistically significant (*p*-interaction = 0.46 for SBP and 0.19 for DBP). Cord blood lead was not associated with SBP or DBP. There were no significant interactions between sex and cord blood lead.

**Table 3:** Adjusted difference [β-coefficient (95% CI)] in child BP for an IQR increase in maternal bone (*n* = 457) and cord blood lead (*n* = 323), overall and by sex.

Measure	Tibia lead (IQR = 13 μg/g)	Patella lead (IQR = 16 μg/g)	Cord blood lead (IQR = 4 μg/dL)
Overall						
SBP		0.96 (–0.13, 2.05)		0.44 (–0.72, 1.61)		0.92 (–0.55, 2.39)
DBP		0.46 (–0.52, 1.33)		0.23 (–0.74, 1.20)		0.92 (–0.11, 1.95)
Girls						
SBP		2.11 (0.69, 3.52)**		0.87 (–0.75, 2.49)		0.75 (–1.13, 2.63)
DBP		1.60 (0.28, 2.91)*		0.83 (–0.66, 2.31)		0.96 (–0.22, 2.15)
Boys						
SBP		–0.34 (–1.98, 1.30)		0.01 (–1.64, 1.65)		1.23 (–1.11, 3.56)
DBP		–0.83 (–2.05, 0.38)		–0.38 (–1.56, 0.79)		0.84 (–0.91, 2.59)
All models were adjusted for maternal education; smoking during pregnancy; dietary intakes of calories, calcium, and iron during pregnancy; infant birth order; gestational age and weight at birth; and child age, height, and BMI at the time of BP measurement. *p = 0.007, **p = 0.025, derived from cross-product terms of sex and tibia lead.						

Additional adjustment for concurrent blood lead, in the sample with those measurements available (*n* = 367), changed parameter estimates for maternal tibia lead by about 2% and 17.5% for SBP and DBP, respectively (Table 4), suggesting that concurrent blood lead is a negative confounder. The interaction between sex and tibia remained significant for DBP (*p*-interaction = 0.006) but was only marginally significant for SBP (*p*-interaction = 0.08). Further adjustment for maternal DBP reduced the effect estimate by 32%, as expected (data not shown).

**Table 4:** Associations between prenatal lead exposure and BP further adjusted by current lead exposure.

Measure	Tibia lead (IQR = 13 μg/g)	Patella lead (IQR = 16 μg/g)	Cord blood lead (IQR = 4 μg/dL)
Overall						
SBP		1.22 (0.02, 2.42)		0.24 (–1.08, 1.55)		0.71 (–0.87, 2.30)
DBP		0.64 (–0.41, 1.68)		–0.02 (–1.07, 1.04)		0.86 (–0.21, 1.92)
Girls						
SBP		2.15 (0.62, 3.67)**		0.49 (–1.40, 2.38)		0.74 (–1.18, 2.67)
DBP		1.88 (0.45, 3.34)*		0.32 (–1.34, 2.0)		0.84 (–0.40, 2.09)
Boys						
SBP		0.04 (–1.82, 1.90)		0.02 (–1.84, 1.81)		0.88 (–1.03, 2.80)
DBP		–0.96 (–2.35, –0.44)		–0.37 (–1.59, 0.85)		0.65 (–1.97, 3.27)
Parameter estimates represent the difference in child BP (mmHg) for an IQR increase in maternal bone (*n* = 367) and cord blood lead (*n* = 323), overall and by sex. All models adjusted as in Table 3 and with additional adjustment for child’s concurrent blood lead level at the time of BP measurement. *p = 0.005, **p = 0.08, derived from cross-product terms of sex and tibia lead.						

**Figure 1:**
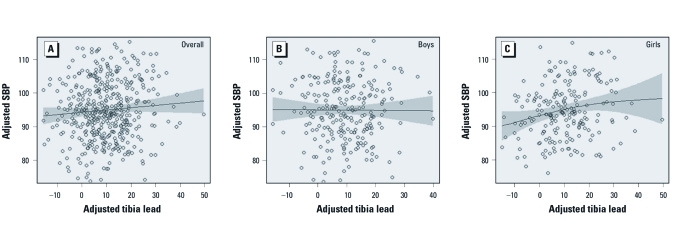
Partial residual plots of systolic blood pressure (SBP) and tibia lead levels, and smoothed terms (regression curve and 95% CI) derived from additive models for overall group (*A*), boys (*B*), and girls (*C*), adjusted for maternal education, smoking during pregnancy; dietary intakes of calories, calcium, and iron during pregnancy and infant birth order; gestational age and weight at birth, and child age, height, and BMI at the time of BP measurement.

Partial residuals of BP and tibia lead and the smoothed associations, overall and stratified by child sex, are shown in Figure 1. There were no deviations from linearity among the overall group or among males. Although there appears to be a slight curvilinear relationship between tibia lead and SBP among girls, the association did not deviate from linearity (*p* = 0.09). The data are thus consistent with a linear dose response among females.

## Discussion

This is the first study to examine the association of maternal bone lead, as a marker of prenatal exposure, with child BP. We examined children’s BP, which is an established precursor of hypertension, atherosclerosis, and left ventricular hypertrophy in adulthood (Daniels et al. 1998; Lauer and Clarke 1989; Litwin et al. 2006) and also assessed the potential role of sex in modifying the response to prenatal lead exposure. In this prospective follow-up study, we observed positive associations between maternal tibia lead, not patella or umbilical cord blood lead, and both SBP and DBP among girls only. These sex-specific associations were independent of child’s gestational age and weight at birth, birth order, age, and BMI and were not affected by maternal smoking or dietary intakes of calories, calcium, or iron during pregnancy.

It has long been known that the prevalence of hypertension and CVD differs between men and women (McBride et al. 2005). However, a gap in knowledge remains regarding the etiology of risk differences between the sexes (Pilote et al. 2007). Although genetic background and lifestyle may contribute, these factors do not fully explain the sex dichotomy in disease susceptibility.

Females may be at disproportionate risk to the developmental programming effects of prenatal exposure to lead. The association of tibia lead with child DBP in this study was attenuated after adjusting for maternal DBP, suggesting that maternal accumulated lead exposure may also program sex-specific adaptive responses via a maternal BP influence. Because pregnant women with chronic or gestational hypertension were excluded from our study, and their children may be at higher risk for higher BP, the associations of prenatal lead exposure with child BP could be underestimated in this study.

Our findings, if confirmed in larger studies, indicate that higher bone lead in women not only may result in increased risk of hypertension in the women themselves (Korrick et al. 1999) but may also affect the subsequent cardiovascular health of their daughters. Our study highlights the need, besides continuing efforts to eliminate lead from and prevent lead release into environment, for secondary preventive measures, such as dietary calcium supplementation (Ettinger et al. 2009), to reduce skeletal lead accumulation and resultant internal exposure through bone lead mobilization, especially in women of reproductive age.

One of the unique advantages of this study is that we measured maternal bone lead as a marker of cumulative lead exposure over the course of pregnancy. Levels of lead in maternal tibia (9.3 μg/g) and patella (11.6 μg/g) in our subjects were similar to bone lead levels reported previously in Mexican-American women (Rothenberg et al. 2002). In contrast to umbilical cord lead, which represents exposure to the fetus around the time of delivery, maternal bone lead represents antenatal lead exposure even before conception. Lead accumulates in bone with a half-life on the order of years to decades and can persist many years after external sources of exposure have declined (Barbosa et al. 2005; Hu 1998). Substantial fetal lead exposure can occur from mobilization of cumulative maternal skeletal lead stores into the circulation during pregnancy (Hu 1998; Tellez-Rojo et al. 2004).

Tibia lead was more strongly associated with SBP and DBP than was patella lead. The patella consists mostly of trabecular bone and thus has a higher turnover rate than does the tibia, which consists mainly of cortical bone and has a longer half-life with respect to lead, and therefore better represents accumulated exposure. Bone turnover is considered the major source of circulating lead in absence of ongoing external sources of exposure (Hu et al. 1998). Thus, patellar lead would be expected to exert the greatest impact on BP if the mechanism required only mobilization of bone lead stores during pregnancy. The observed association of long-lived tibia lead with girls’ BP leads us to speculate that the sex-specific lead impacts on adult disease programming might begin quite early in germ cell development and fetal life. Both our study and the Oswego Children’s Study (Gump et al. 2005) have revealed associations in children, but with different lead exposure biomarkers (e.g., tibia lead vs. cord blood lead). In contrast to the Oswego Children’s Study, we found an association between children BP and tibia lead but not with cord blood lead. The reason for this inconsistency may be attributable to differences in children’s ages and study locations and design, or adjustment for additional factors such as maternal depression or prenatal exposures to mercury and pesticides that was not done in the present study. It is also noteworthy that the mean (± SD) cord blood lead in the present study (5.51 ± 3.45 μg/dL) was nearly three times higher than that seen in Oswego Children’s Study (1.98 ± 1.75 μg/dL). Further studies are needed to clarify the apparent discrepancy.

The biological pathway(s) that may underlie sex differences in the impact of prenatal lead exposure are not clear, but clues may be gained from known lead toxicity mechanisms in adults. Compared with boys and age-matched men, girls and premenopausal women have been reported to have lower BP and hypertension prevalence, but such differences disappear when women reach menopause (Dasgupta et al. 2006; McBride et al. 2005; Pilote et al. 2007), suggesting that ovarian estrogen steroids, via their roles in neural and hormonal regulations of BP, may protect women from hypertension and CVD. An impact of ovarian estrogen hormones on lead toxicity has been observed in studies where postmenopausal women were found to be more sensitive to hypertensive effects of lead than were premenopausal women (Korrick et al. 1999; Nash et al. 2003; Potula and Kaye 2006). Early stages of fetal development have more plasticity than during any other time in life and are sensitive to maternal–fetal stressors that can interfere with the natural hormones, neurotransmitters, and growth factors controlling development. Lead has been shown to interfere with estrogen metabolism by direct ovotoxicity and via indirect effects on the hypothalamus–pituitary–ovarian axis (Hoyer 2005). Prenatal exposure to lead, at environmentally relevant concentrations, has been found to decrease circulating estrogen levels in adult rats (Dearth et al. 2002). It is biologically plausible that girls may be more sensitive to the hypertensive effects of prenatal lead exposure because of the interruption of estrogen metabolism.

Recently, our group reported that prenatal lead exposure is associated with decreased genomic DNA methylation (Pilsner et al. 2009). The consistent changes in the DNA methylation of some imprinted and growth-promoting genes have also been found to be associated with prenatal famine in humans, which depend on the sex of the child and gestational timing of the exposure (Tobi et al. 2009). As such, it is possible that the resulting epigenetic alterations may change developmental estrogenization and sex-specific susceptibility throughout the life course (Gabory et al. 2009). Because hypertension is a polygenetic disorder, innate sex differences in the genetic susceptibility to lead toxicity, in addition to sex hormone–mediated attenuation of other sex-related common determinants, may also be involved in developmental programming (Gilbert and Nijland 2008). Further research is needed to examine whether those mechanism(s) are responsible for the apparent association of prenatal lead exposure with BP in females.

The present study has several limitations. Despite the standardized protocol, the single measure of BP using a mercury sphygmomanometer is prone to measurement error. Such errors are random, however, and promote attenuation of observed effects rather than the generation of spurious associations. Children’s BP is known to rise progressively with chronological age and body size and more rapidly in puberty (Leccia et al. 1999). It is possible that the observed impact of bone lead on BP in girls may be, in part, mediated by variation of age of onset of puberty, with girls reaching puberty 3 years earlier, on average, than boys. As described above, the birth cohort study conducted only one follow-up visit after early childhood (7–15 years) and was not specifically designed to assess sexual maturation. Therefore, the age of onset of puberty was not known. We examined this possibility by comparing impacts of maternal bone lead on child BP between girls 10–11 years of age and boys 13–14 years of age and found that the apparent sex difference within all participants was not substantially changed (data not shown). Previous studies indicate that lead levels as low as 3 μg/dL, and independent of body size, delay growth and pubertal development in girls (Selevan et al. 2003). Therefore, lead would likely be associated with decreased rather than increased BP, which is opposite to our findings. Our study and others noted here do not support puberty as a potential confounder between lead and BP. However, we cannot rule out such a possibility based on these data. Assessments of sexual maturation are needed in future studies to delineate the true sex differences. In addition, because child’s diet and physical activities were not measured, we could not determine whether the effects of bone lead are related to those general lifestyle factors. Finally, because our study was conducted in a Mexican population, the results may be not applicable to non-Hispanic ethnic groups.

## Conclusion

Our results suggest that long-lived maternal bone lead stores acquired from previous environmental lead exposure pose a risk of higher BP in girls but not boys. Thus, differences in BP observed between women and men across young and middle age may stem, at least partially, from increased likelihood of lead exposure during early-life development. Because elevated BP in childhood is a known risk for hypertension in adulthood, continuing follow-up of these children over the years is warranted.
